# Transient Characteristics of a Fluidic Device for Circulatory Jet Flow

**DOI:** 10.3390/s18030849

**Published:** 2018-03-13

**Authors:** Hoa Thanh Phan, Thien Xuan Dinh, Phong Nhu Bui, Van Thanh Dau

**Affiliations:** 1 HaUI Institute of Technology, Hanoi University of Industry (HaUI), Hanoi 100000, Vietnam; 2 Graduate School of Science and Engineering, Ritsumeikan University, Kyoto 525-8577, Japan; thien@cfd.ritsumei.ac.jp; 3 Faculty of Electronic Engineering, Hanoi University of Industry (HaUI), Hanoi 100000, Vietnam; phongbn.haui@gmail.com; 4 Research Group (Environmental Health), Sumitomo Chemical. Ltd., Hyogo 665-8555, Japan

**Keywords:** transient characteristics, circulatory flow, 3D simulation, OpenFOAM, confined space

## Abstract

In this paper, we report on the design, simulation, and experimental analysis of a miniaturized device that can generate multiple circulated jet flows. The device is actuated by a lead zirconate titanate (PZT) diaphragm. The flows in the device were studied using three-dimensional transient numerical simulation with the programmable open source OpenFOAM and was comparable to the experimental result. Each flow is verified by two hotwires mounted at two positions inside each consisting chamber. The experiment confirmed that the flow was successfully created, and it demonstrated good agreement with the simulation. In addition, a prospective application of the device as an angular rate sensor is also demonstrated. The device is robust, is minimal in size, and can contribute to the development of multi-axis fluidic inertial sensors, fluidic amplifiers, gas mixing, coupling, and analysis.

## 1. Introduction

An enclosed flow system that features a minimum amount of samples, enhances mixing, and is partly free from the environment is considered an essential microfluidic device [1,2,3,4]. In an enclosed system, flow circulates and increases the capability of microfluidic systems. A circulatory jet flow in a confined space might find most of its applications in, besides fast flow control [5] and the cooling of high power devices [6], inertial sensing because of the absence of moving parts and the minimal effect from linear acceleration [7,8], thus reducing the risk of mechanical damage [9]. 

One of the methods to create such flow consists in vibrating the liquid with a piezoelectric diaphragm (PZT) to rectify flow into a jet with the average velocity of several meters per second [10,11]. Another approach includes releasing jet flow using electrohydrodynamic force from a high electric field to activate an electro-conjugate fluid [12,13,14]. A third method is natural convection from a locally heated region, which follows the direction of mass diffusion [15,16,17,18,19,20]. Slightly different from natural convection, thermal expansion from a quickly heated gas has recently been used to move the fluid. The thermal expansion is dominant in the region near the heating center at the initial state with a high-temperature gradient; when the temperature gradient decreases as the as the gas expands further, the flow becomes the natural convective and depends on gravity [21,22,23,24].

The main advantage of a system with a circulatory jet flow is the forced convection, which is more robust with gravity. In this context, a laminar jet flow is usually synthesized by vibration from a PZT diaphragm, although other vibrating actuators are also widely used [25,26]. In studies of the integration of synthetic jets with a PZT device, numerical and analytical models have been presented in order to determine the volume flow rate for open systems described by the far-field boundary condition [27,28,29]. Pumping fluid at a high velocity in a closed loop within a limited space still requires an appropriate rectifying mechanism to overcome the choking phenomenon of the liquid. In addition, due to limited access to the flow, the enclosed system has some specific practical challenges in design and evaluation [30]. Therefore, many designs do not mention the flow-generating parts, or they merely rely on an external flow source [31,32].

In our previous work, simple configurations of a single-and dual-axis fluidic gyroscope were developed [33,34]. However, the device made by conventional machining techniques involves a sub-assembled PZT pump. The improved design with a laminar jet flow was introduced later, but the study was limited by the fundamental design, and experimental work was not sufficiently evaluated to achieve the desired results [35,36,37,38]. Several concepts have been introduced by other research groups, such as PZT pumping integrated with a microfabrication process in the fluidic gyroscope, but these studies lacked experimental investigation [20,39] and pumping integration [40].

In this study, we present the experimental study of a piezoelectrically actuated microfluidic device that can circulate multiple jet flows. The design and working principle of the device are described, followed by transient flow simulations and experimental validation. The symmetrical characteristic of flow is also investigated using a turntable, and the effect of several governing parameters was also discussed.

## 2. Design and Numerical Simulation

**Figure 1:**
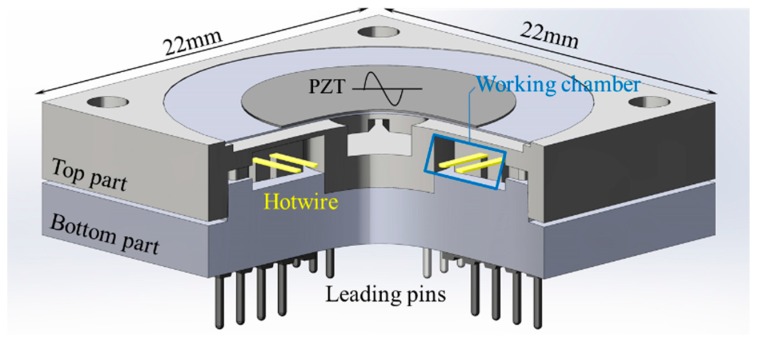
Cut view of a circulatory flow device. The device is completely sealed, and its internal flow in the working chamber is measured by the voltage on the implemented hotwire, through the leading pins.

**Figure 2:**
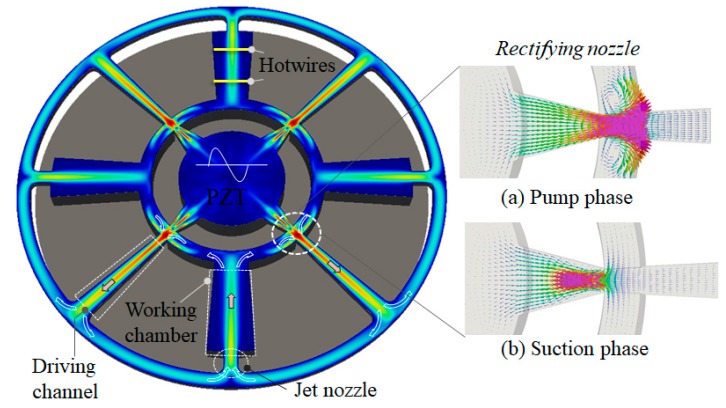
Time-averaged flow fields inside the device (red: highest velocity). The arrows indicate flow direction. (**a**) The insets show instantaneous flow in the pump phase where most of the flow goes straight out of the pump chamber entering the driving channel. (**b**) In the suction phase, the drawn back flow is mostly from the spaces perpendicular to the driving channel.

The conceptual design of the device has been introduced in previous work and is summarized as follows. The device consists of a flow network and a PZT diaphragm on the top, which is sealed from the ambient environment, as shown in Figure 1. The fluidic network is symmetrical around its center axis and has four working chambers and four driving channels. Each working chamber is connected by two driving channels at the outermost radius and by the PZT chamber at the center of the device. The PZT chamber is in a disc form with a diameter of 18 mm and has a depth of 0.3 mm. The driving channel centrifugally diverges in the fan-shape with an open angle of 6° and a depth of 0.5 mm. The working chambers are centripetally converged with an angle of 12° and a depth of 1.5 mm. In the working chamber, the flow is shaped into jet form by a jet-forming nozzle in accordance with the geometry of the chamber (Figure 2).

To validate the flow characteristics, in each working chamber, two hotwires are implemented at distances of 1 and 2.5 mm from the nozzle. The hotwires are centered, crossed over the working chamber, and led out by conventional lead pins embedded in the bottom of the device’s body.

The working principle and flow measurement are described by transient simulations in a three-dimensional model for laminar flow. Because the flow is self-generated in a closed space, the fluid is treated as compressible medium governed by the following equations:

(1) $$\frac{\partial \rho }{\partial t}+\nabla \cdot \rho \vec{u}=0$$

(2) $$\frac{\partial \rho \vec{u}}{\partial t}+\left(\vec{u}\cdot \nabla \right)\rho \vec{u}=-\nabla p+\nabla \cdot \left(\mu \nabla \vec{u}\right)$$

(3) $$\frac{\partial \rho c_{p}T}{\partial t}+\left(\vec{u}\cdot \nabla \right)\rho c_{p}T=\nabla \cdot \left(\lambda \nabla T\right)$$

where $\vec{u}$, *p*, and *T* denote the velocity vector, pressure, and temperature of the flow field, respectively. As air is the working fluid, the physical properties are $\mu =1.789\times 10^{-5}\;\mathrm{Pas}$, $\rho =1.2041\;\mathrm{kg}\cdot m^{-3}$, $\lambda =2.42\times 10^{-3}\;W\cdot m^{-1}\;K^{-1},$ and $c_{p}=1006.43\;J\cdot {\mathrm{kg}}^{-1}K^{-1}$ denoting the dynamic viscosity, density, thermal conductivity, and specific heat, respectively. The relationship between the pressure and density follows the state equation $p=\rho R_{u}T/M_{w}$, where $R_{u}=8.314\;J\cdot {\mathrm{mol}}^{-1}K^{-1}$ is the universal gas constant and $M_{w}=28.96\;g\cdot {\mathrm{mol}}^{-1}$ is the molecular weight.

For the boundary conditions, the local rate of deformation of the PZT diaphragm is converted to a local velocity and is applied to the diaphragm surface as follows:

(4) $$v\left(\vec{r},t\right)=2\pi fZ\cos\left(2\pi ft\right)\varphi \left(\vec{r}\right)$$

where the deformation shape φ(r) can be expressed as $\varphi \left(r\right)={\left(1-{\left(r/a\right)}^{2}\right)}^{2}$, *a* = 10 mm is the radius, and *Z* is the central deflection of diaphragm. 

The deflection amplitude, Z, was measured by a laser Doppler in [41]. The simulation is performed using OpenFOAM with the time step determined as one-twentieth of the PZT diaphragm vibration period. The simulation domain was modeled with a hexagonal mesh clustered near the wall. A fine mesh step was used in the nozzle to describe a high velocity change of the flow field; in particular, a minimum dimension at the nozzle was controlled with 10 meshing divisions. For solver setting, the semi-implicit method for the pressure-linked equation is engaged and the second-order upwind scheme is used for spatial discretization of the momentum, density, and energy equations. The calculation is advanced with time by the implicit second-order scheme. 

**Figure 3:**
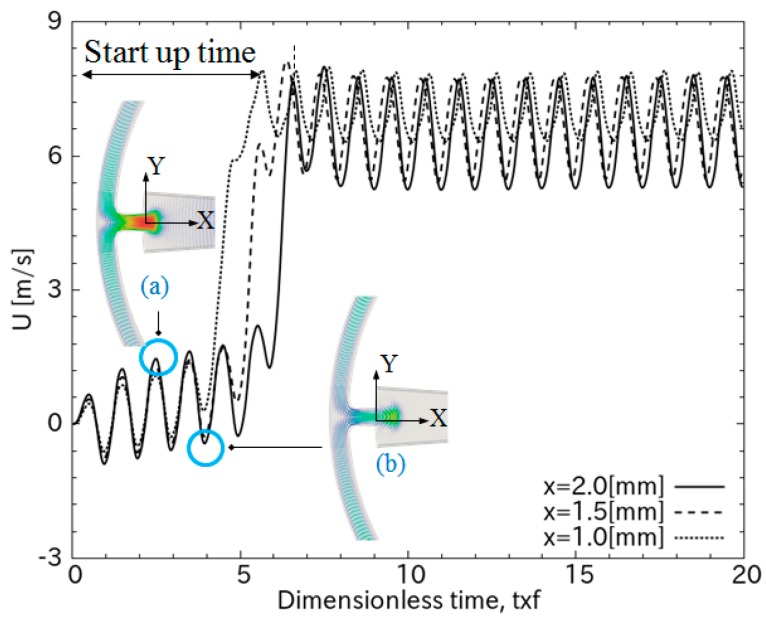
Transient characteristic of the flow in working chamber. The insets show jet flow at the nozzle entering the working chamber in (**a**) the pumping phase and (**b**) the suction phase. The amplitude of diaphragm deflections is 10 μm.

Figure 2 shows the flow generation principle demonstrated by simulation. In the pump phase when the PZT diaphragm moves down to compresses the pump chamber, the air in the pump chamber is pushed straight into the driving channel (Figure 2a). In the suction phase, the diaphragm moves up, the pump chamber expands, and the backflow in the driving channel slows down because of the interruption of the flows from the spaces perpendicular to the driving channels. As a result, a net flow is rectified in the driving channel and propagates to the working chamber (Figure 2b). In the view of the transient characteristic, these pump and suction phases match with the peak velocities at each cycle, as shown in Figure 3.

As a small net flow propagates from the pumping channel to the working chamber and circulates back to the rectifying nozzle, its momentum dramatically amplifies the rectifying effect of the nozzle. This is indicated by the sudden increase in average flow velocity after start-up time (transient time) in Figure 3, which shows the transient result at several positions downstream from the nozzle in the working chamber. After several circulations, the flow velocity reaches a quasi-steady state. Its velocity can be of the order of several meters per second as it expands through the working chamber. Figure 3 also depicts that the flow periodically oscillates in each cycle of *t* × *f*, indicating that the flow vibrates with the same frequency as the PZT diaphragm. 

**Figure 4:**
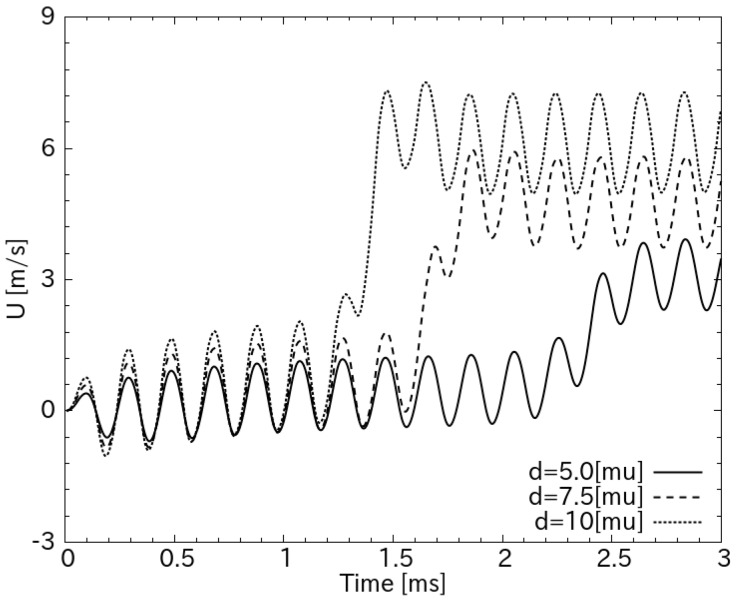
Transient characteristic of the flow in the working chamber with different amplitudes of the diaphragm. The flow is monitored at 2.5 mm downstream from the nozzle. The amplitudes of the diaphragm are 5 μm, 7.5 μm, and 10 μm.

Figure 4 shows the transient characteristic of the average flow in the working chamber with different amplitudes of the PZT diaphragm. The start-up time required for a quasi-steady state is a function of the amplitude of diaphragm deflection. Increasing the deflection amplitude makes the flow travel faster and the start-up shorter. In Figure 4, it is shown that the transient time is almost inversely proportional to the amplitude. The start-up time reduces from 3 ms for d = 5 μm to less than 2 ms for d = 10 μm.

**Figure 5:**
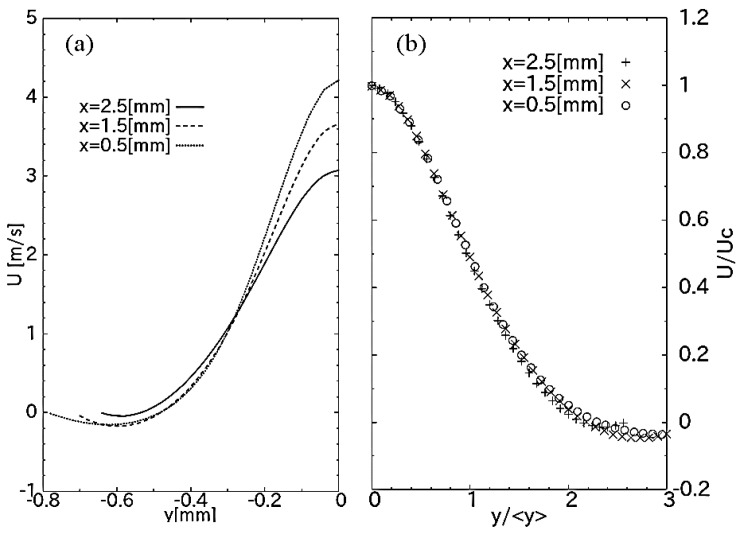
(**a**) Velocity profiles and (**b**) self-similarity of the jet flow at different axial distances *x* from the nozzle. The amplitude of diaphragm deflections is 5 μm.

The velocity profile of axial velocity *u*(*x*, *y*, *z* = 0) is plotted in Figure 5a for the case of diaphragm deflection 5 μm (coordinate presented in Figure 3). While its peaks decrease with an increase in distance *x* from the nozzle, the flow retains a jet profile. In addition, as the central characteristic of a free jet is usually self-similar, we may predict the freedom of a jet flow in a space by examining its self-similarity. With the center line of axial velocity *U_C_* = *u*(*x*, 0, 0), the jet’s half-width <*y*> is defined as the transverse distance from the jet axis to the location at which the mean velocity *U*(<*y*>) = ½ *U_c_* [42]. The axial velocity is plotted again in Figure 5b, where the vertical axis is the flow velocity scaled to the corresponding peak values and the abscissa is scaled by the transverse distance *y*/<*y>*. All the scaled profiles fall into a single curve, indicating that the jet flow in the chamber possesses self-similarity. It also implies that the jet flow freely moves in three dimensions in the range of 0.5 mm < *x* < 2.5 mm. This is a principal factor in applying this device for inertial sensing application since the sensing principle is inherited from the axisymmetric property of the free jet flow. 

## 3. Experimental Setup 

**Figure 6:**
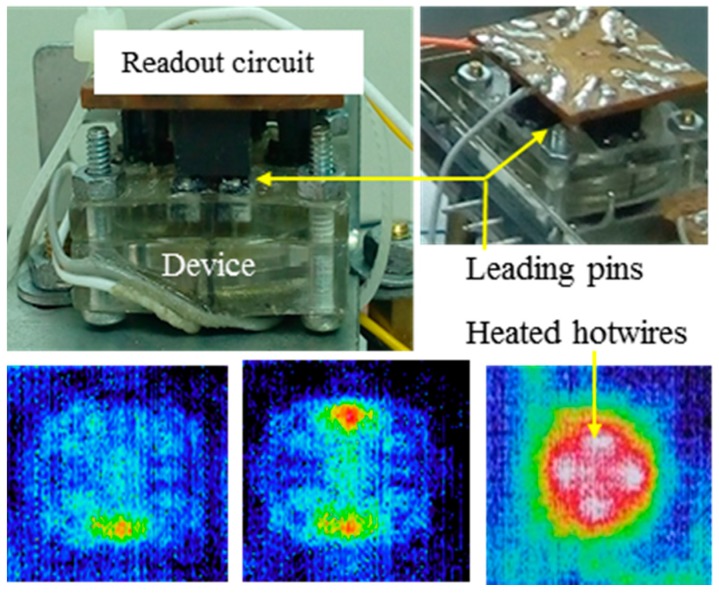
Experimental setup to confirm flow characteristics. The device was packed and connected to a readout circuit. The insets show a thermal image of the heated hotwires in each chamber before the device was packed (red: highest temperature).

For the experiment, the device was made using polymethyl methacrylate (PMMA) with a mechanical precision of 20 µm (Figure 6). The device is closed by sealing the PZT diaphragm [43] on top of the pump chamber. The fabricated device contained total dimensions of 21 mm × 21 mm × 2.5 mm (width × length × thickness). Two kinds of hotwires, gold and tungsten, were implemented for the test. The gold hotwire had a diameter of 20 μm, a temperature coefficient of resistance (TCR) of 3700 ppm/°C, and was directly bonded on the lead pins. The tungsten hotwire, a diameter of 10 μm, and a TCR of 4500 ppm/°C was conductively glued to the lead pin. The length of the hotwires was 3.0 mm.

Before the device was packed, the heating of the hotwires was confirmed using a thermal camera Avio H2640 (Nippon Avionics Co. Ltd., Tokyo, Japan). The inset shows the thermal image of the device when the hotwire in each working chamber was heated with constant current *I* = 30 mA. 

The PZT was driven by a sinusoidal signal from a function generator IWATSU SG-4105 (Iwatsu Electric Co. Ltd., Tokyo, Japan). A constant current was applied to the hotwire and its voltage was read via a TEXIO DC power supply PW18-1.8AQ (Texio Co. Ltd., Kanagawa, Japan). For time-resolved measurement, the data was streamed to the computer using a SignalExpress on NI9234 device (National Instruments Corporation, Austin, TX, USA) with a sampling rate of 25.6 kHz. The ambient temperature was maintained at standard room conditions, 22–25 °C. 

## 4. Experimental Results and Discussion

### 4.1. Pumping Performance Confirmed by Hotwire Anemometry

**Figure 7:**
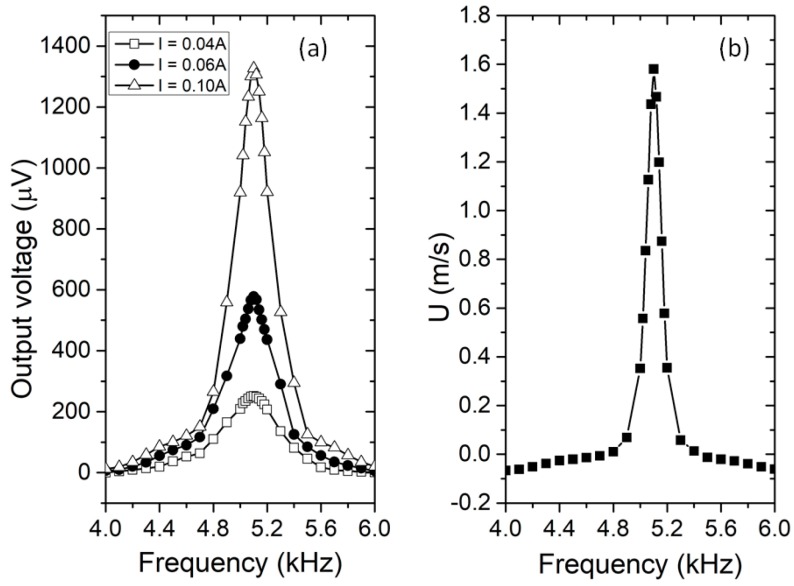
Frequency response of the device. The actuating voltage on PZT is 8 V. The tungsten hotwire was heated using different currents and the measured flow velocity was converted from hotwire output voltage (*I* = 0.1 A).

The flow characteristic of the device was investigated using hotwires as flow sensing. In this work, the hotwire was heated by a constant current *I* = 40 mA, 60 mA, and 100 mA. When the PZT diaphragm vibrated, the flow cooled the hotwire down by forced convection and the relative voltage on the hotwire was directly measured as $V_{HW}=I\Delta R_{HW}=I\alpha \Delta T,$ where *α* is the temperature coefficient of resistance of the hotwire material. This voltage is shown in Figure 7 as a relationship with the frequency of the applied voltage, *V_PZT_* = 8 *V_pp_*, on the diaphragm. The output from the hotwire reached its peak at a frequency of 5.1 kHz. This behavior follows the frequency response of the diaphragm at its resonant vibration, indicating that the interaction from the air damping to the stiffness of the diaphragm is small. Figure 7a also shows a general increase in *V_HW_* with the heating current. The increase in output voltage is due to both Ohm’s law with a larger current and a stronger heat transfer process. Therefore, the heating current can be used as a governing parameter to adjust the working range of the device.

The resistance variations of hotwires were then converted to the velocity of ion wind by comparing the heat transfer coefficient of forced convection, $0.24+0.56Re^{0.45}\lambda /D,$ from generated ion winds with that of natural convection $1.02Ra^{0.1}\lambda /D\;$ from a still air [44], where λ  is the thermal conductivity, *Ra* is the Rayleigh number, *d* is the effective diameter of the hotwire, and Re=UDρ/μ is the Reynolds number. The process was carried out using an subroutine C-code, which has been detailed in our recent publications [45,46,47,48]. The average velocities calculated from output voltage are plotted in Figure 7b. It is noted that, because the hotwire was placed across the entire width of the working chamber, its measurement represents the cooling effect of the space-averaged flow velocity in the hotwire position, which reaches 1.67 m/s at the resonant frequency. When the diaphragm deflection at *V_PZT_* = 8 *V_pp_* is 4.8 μm (in Section 4.2 below), this measured space-averaged velocity relatively matches the simulation result in Figure 4, which shows the peak velocity at the central axis of the working chamber.

**Figure 8:**
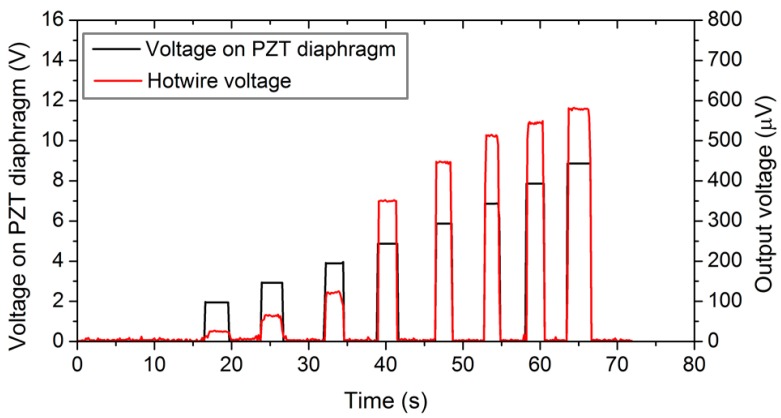
Change in hotwire voltage with increasing pump voltage.

Figure 8 plots the variation of measured *V_HW_* as a function of the driving voltage, *V_PZT_*, actuating at the resonant frequency and heating current, *I* = 60 mA. The output of the hotwire *V_HW_* increases with increasing driving voltage. This is obvious since a larger *V_PZT_* creates a large deformation of the diaphragm and produces a stronger flow velocity, as seen in Figure 4. The time response of the output voltage in relation to the driving voltage was also recorded and was used to determine the transient characteristics of the device, as shown in the section below.

### 4.2. Transient Characteristic of Flow

**Figure 9:**
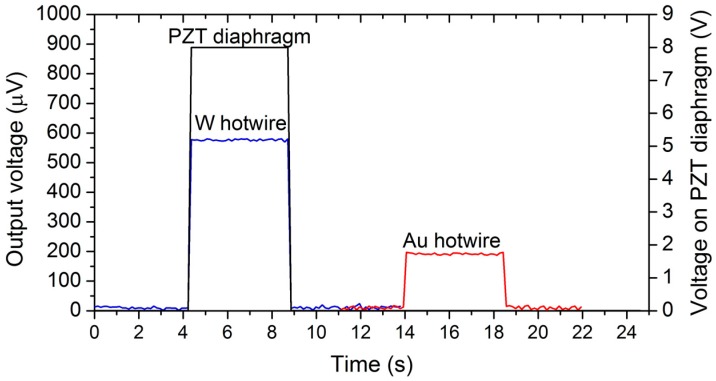
Hotwire voltage changes between gold hotwire and tungsten hotwire and the time-resolved data between actuating voltage and output voltage. The data corresponding to the gold hotwire was independently added to the same *x*-axis for clarification.

Figure 9 shows the time-resolved data between the applied voltage, *V_PZT_* = 8 V_pp_, and output voltage for the gold and tungsten hotwires. As expected, the tungsten hotwire has a much higher output voltage due to its higher TCR and resistivity. The sudden increase in the hotwire voltage indicated that the flow steeply reached its maximum velocity. This result agrees well with the working principle illustrated in Figure 3, whereby the feedback flow from the working chamber boosts the effect of the rectifying nozzle.

**Figure 10:**
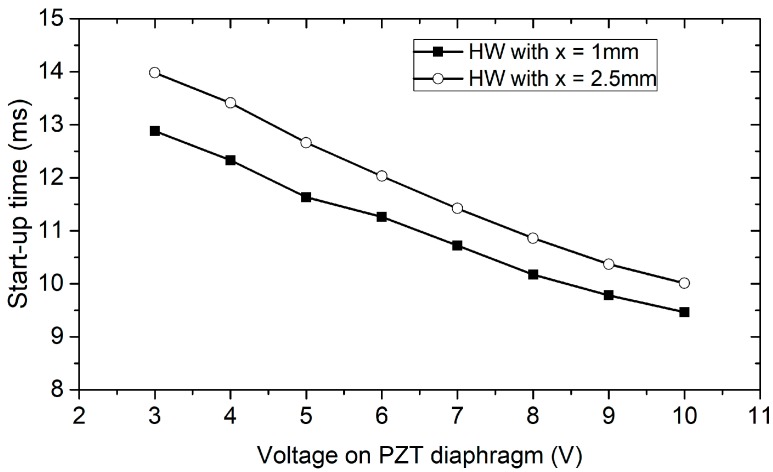
Relationship of the start-up time, measured at hotwire positions of 1 mm and 2.5 mm and driving voltage on the PZT diaphragm. The diaphragm was actuated at the resonant frequency.

The start-up time of the device was acquired from the transient time of the output voltage and is plotted as a function of applied voltage *V_PZT_* in Figure 10. It is shown that the start-up time was inversely proportional to the driving voltage or, in other words, the amplitude on the diaphragm. For the hotwire located 1 mm from the nozzle, the start-up time was 13.98 ms and 9.76 ms with an applied voltage of *V_PZT_* = 3 V and 10 V, respectively, at 30 mA of the hotwire current.

The conversion between the applied voltage and the deformation of this PZT diaphragm was measured with a laser Doppler vibrometer LV-1800 (Ono Sokki Co.Ltd., Yokohama, Japan) [41]. In the range of *V_PZT_* <20 V at its resonant frequency of 5.1 kHz, the center deformation is expressed as a linear relation with *V_PZT_* as $Z=0.6V_{PZT\left(V\right)}\pm 0.1$. For the applied voltage in Figure 10, the deformation is from 1.8 to 6 µm. In this range, the measured start-up time is longer than that of the simulation, which is less than 5 ms. The slight difference might come from the effect of the imperfection in device geometries and the actual delay in the electronic circuit. 

Figure 10 also shows a good correlation between the start-up times of the two hotwires, placed 1 and 2.5 mm downstream of the working chamber. The time delay, Δt, between the two hotwires was shorter at a high voltage on the PZT and was lengthened when the driving voltage decreased. At *V_PZT_* = 3 and 10 V, the delay in the start-up time of the two hotwires was 1.1 and 0.54 ms, respectively. Assuming that the two hotwires are identical, the average velocity of flow traveling a distance Δx between the two hotwires can be calculated simply by dividing the corresponding distance with Δt; therefore, U=Δx/Δt and was 1.36 to 2.77 m/s for *V_PZT_* increasing from 3 and 10 V, respectively, which is in good agreement with the simulation, as shown in Figure 4. 

**Figure 11:**
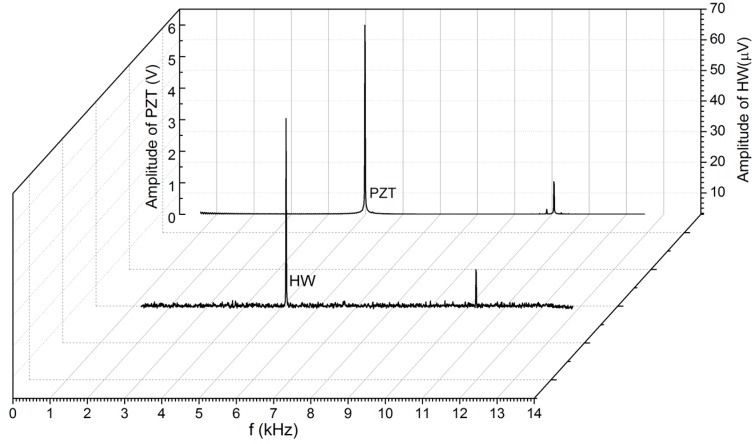
Frequency spectrum of output voltage on the hotwire and driving voltage on the PZT and the relationship between start-up time and different oscillating frequencies at various hotwire currents.

The cooling effect of the jet flow on the hotwire depends on both the frequency response of the jet flow and hotwire. However, the frequency response of the hotwire is very fast. Typically, the frequency response of a hotwire is up to *f* – 1000*f*_a_, where 2*πf*_a_ ≈ 40*D_f_*/(2*l*)^2^ [49,50]. For our tungsten hotwire *l* = 3 mm and the thermal diffusivity *D_f_* – 6.5 × 10^−5^ m^2^·s^−1^, the frequency response of the hot wire is approximately 46 kHz. This response is much faster than that of the flow, which should vibrate at the same frequency of the diaphragm predicted by simulation in Figure 3. This was confirmed by the frequency spectrum analysis of the measured output voltage shown in Figure 11. When the PZT was actuated at a resonant frequency of 5.1 kHz, the spectrum revealed that the peak output voltage of the hotwire is also 5.1 kHz. 

**Figure 12:**
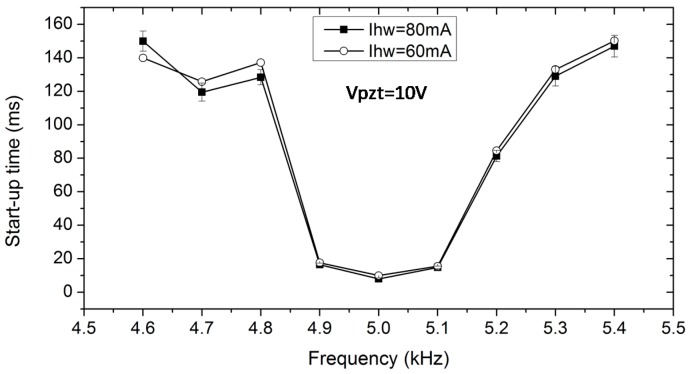
Relation of start-up time versus driving frequency on the PZT diaphragm.

Figure 12 depicts another aspect of the start-up time in relation to oscillating frequencies and hotwire currents. The transient time of the output voltage in Figure 12 is plotted for the hotwire placement, *d_x_* = 2.5 mm, downstream of the working chamber. The start-up time has an inverse relationship with the oscillating frequency of the PZT diaphragm. At a hotwire current of 80 mA, the start-up reached its smallest value when the diaphragm vibrated at its natural frequency. Figure 12 also shows that start-up time is almost independent of the heating current, which can be explained by the fast response of the hotwire. Indeed, the dependence of the start-up time proved that air moving through the sensor chamber was affected by the oscillating frequency of the PZT diaphragm. Thus, an important parameter in designing the device is the PZT diaphragm, which has been selected based on its commercial availability in this work [43].

### 4.3. Application in Inertial Sensing

**Figure 13:**
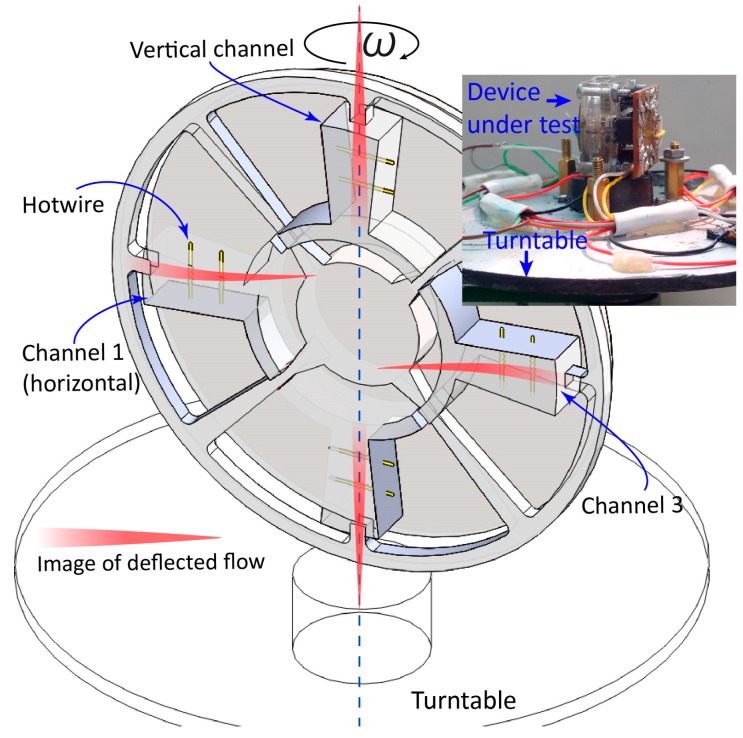
Schematic view of flow inside working chamber deflected under angular velocity $\vec{\omega }$. Flows in two horizontal channels are deflected in the opposite direction. The inset shows an experimental setup with the turntable.

With the jet flow confirmed, the device can be used as a microfluidic gyroscope to detect angular rate. Should the device be vertically placed on a turntable, due to Coriolis acceleration, the rotation of the turntable deflects the flows in the working chambers, which are parallel with the table plane. Because the hotwires are placed at the middle plane of the channel depth, the flow deflection results in a reduction in the flow velocities on the hotwires, as shown in Figure 13 below. This leads to a difference in the temperatures of the hotwires and, accordingly, their resistances. On the other hand, the flows in channels normal to the table plane should simply spin around their longitudinal axis and cause no difference in flow velocity at hotwire positions. 

**Figure 14:**
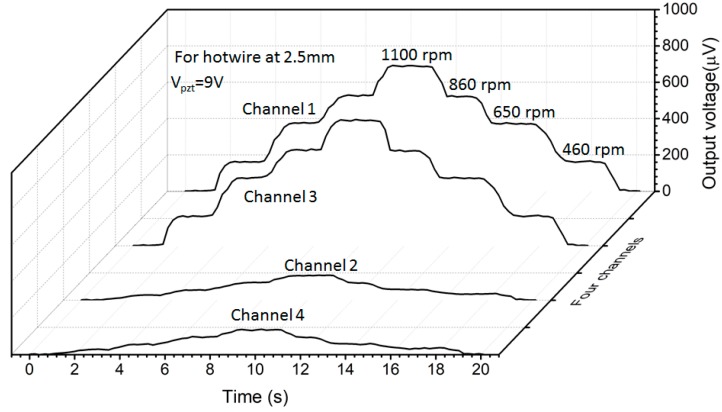
Time-resolved output from hotwires in four channels. The measurement was conducted with *V_PZT_ =* 9 V and a resonant frequency of 5.1 kHz. The voltages are offset from their initial values before the turntable rotates.

Figure 14 shows output voltages in two horizontal chambers parallel with the table plane and in two vertical chambers normal to the table plane. When the table rotates, the voltages on hotwires in the horizontal chambers changes proportionally and their shapes are almost identical, indicating that the flows are similar in these chambers. The scale factor, calculated by the change of output voltage per unit of angular rate, is 0.104 μV·deg^−1^s. Likewise, the output voltage in the vertical chambers is much smaller with a scale factor of 0.006 μV·deg^−1^s. This significantly smaller scale factor demonstrates that the flows in the vertical chamber are self-similar around its axis, so the cooling effect on the hotwire is hardly affected by the spinning of the flow [36]. 

## 5. Concluding Remarks

We here report the design, simulation, mathematical modeling, and experimental analysis of a millimeter-scale device that can generate multiple jet flows circulating in a confined space. The flow was simulated by computational fluid dynamics using OpenFOAM and was verified by experiment employing a hotwire anemometry technique. The simulation and experiment have good agreement. A prospective application as an angular rate sensor is also demonstrated. Additionally, the device itself is easy to build at low cost because of its simple and commercially available components. We believe that our device can contribute to the development of multi-axis fluidic inertial sensors, fluidic amplifiers, gas mixing, and other fluidic applications in micro-mechatronics system.
